# Labour market polarisation revisited: evidence from Austrian vacancy data

**DOI:** 10.1186/s12651-021-00290-4

**Published:** 2021-03-17

**Authors:** Laura S. Zilian, Stella S. Zilian, Georg Jäger

**Affiliations:** 1 Graz Schumpeter Centre, University of Graz, Universitätsstraße 15/F, Graz, Austria; 2 Institute of Systems Sciences, Innovation and Sustainability Research, University of Graz, Merangasse 18, Graz, Austria; 3 Vienna University of Economics and Business, Welthandelsplatz 1, Vienna, Austria

**Keywords:** Skill demand, Polarisation, Network analysis, Vacancies, ESCO, J24, J63, O15, O33

## Abstract

Recent research suggests that new technologies are important drivers of empirically observed labour market polarisation. Many analyses in the field of economics are conducted to evaluate the changing share of employment in low-skill, medium-skill and high-skill occupations over time. This occupation-based approach, however, may neglect the relevance of specific skills and skill bundles, which potentially can be used to explain the observable patterns of labour market polarisation. This paper adds to the literature in two ways: First, we present the results of an analysis of data on job vacancies rather than the currently employed and, second, we derive occupation-defining skills using network analysis tools. The analysis and tool usage allowed us to investigate polarisation patterns in Austrian vacancy data from 2007 to 2017 and identify changes in the skills demanded in job vacancies in Austria. In contrast to most previous research, we find no evidence for polarisation, but rather a trend towards upskilling.

## Introduction

The effects of digital technologies on labour markets worldwide are currently being widely discussed. Several authors predict that these technologies will destroy jobs to various extents due to automation (Arntz et al. 2016; Frey and Osborne 2017), while others stress that they will lead to the emergence of new occupations and the creation of new jobs (Bainbridge 2015) or the transformation of existing jobs (Berger and Frey 2015). Retrospective studies present less dystopian views than future-oriented research regarding the spectre of job destruction due to automation. For example, Graetz and Michaels (2018) demonstrate that the increased usage of robots in 17 countries from 1993 to 2007 in industrial production had a positive impact on productivity and that no overall negative employment effects were observed; however, the employment share of low-skill labour decreased. Nonetheless, the empirically observed polarisation of labour markets (i.e. the increase in the share of low- and high-wage/-skill occupations in the US and the UK) is often explained by the capacity of technologies to substitute for specific work tasks (Autor 2015; Goos and Manning 2007; Goos et al. 2014). These analyses are often based on occupation wages and employment shares (Autor 2015, 2013; Autor and Dorn 2013; Goos et al. 2009, 2014) and the distinction of routine and non-routine tasks as introduced by Autor et al. (2003).

However, several research groups recently indicated that these studies need refinement (e.g. Alabdulkareem et al. 2018; Caines et al. 2017; Salvatori 2018), noting that the traditional occupation-based approaches may neglect the relevance of specific skills and skill bundles, which can potentially be used to explain patterns of labour market polarisation. This aspect is also highlighted by Deming and Kahn (2018) who use vacancy data and exploit the detailed job descriptions to demonstrate that the demand for skills is heterogeneous within occupations, industries and across geographic locations. Hershbein and Kahn (2018) also provide evidence for upskilling of vacancies during recessions.

The latter two studies place a focus on skills, but they also indicate that researchers can analyse labour market polarisation based on vacancy data as a viable alternative to conducting research based on employment data. Most scholars in this field discuss the polarisation of employment patterns for currently employed individuals. While this discussion illustrates shifts that have already taken place (since occupational employment can be considered as the equilibrium outcome of labour supply and demand), an analysis of job vacancies may shed light on emerging trends, providing information about the (unmet) labour demand. We predict that the observable polarisation patterns in employment will also be visible in vacancy data, as technological change is assumed to affect labour market polarisation through its impact on labour demand.

Thus, we applied a promising approach to learn more about the technology-driven polarisation of labour markets by analysing job vacancy data and the skills demanded in vacancies.

This paper contributes to the existing literature in two ways: First, we present the results of an evaluation of labour market polarisation based on Austrian vacancy data and, second, we examine the changes in skills that are demanded to fill these job vacancies based on information derived from the classification of European Skills, Competences, Qualifications and Occupations (ESCO). In particular, we address the following research questions:

- Can we observe polarisation patterns in Austrian vacancy data by performing a skill ranking of vacancies rather than occupational groups?
- Can we observe changes in skills demanded in the Austrian vacancy data?

To answer the first question, we analyse job vacancy data provided by the Public Employment Services Austria (PES) using skill level categories. In contrast to previous research in which wage-based occupational rankings (Goos et al. 2014) or task-based approaches (Autor et al. 2003) were used, we apply the comprehensive skill level classification framework for occupations proposed by the International Labour Organization (ILO 2012). This framework provides four broad skill level categories characterised by several dimensions related to the skill level and skill specialisation needed to perform the tasks in a job.^1^

To answer the second question, we use network analysis tools to connect the ESCO classification to the vacancy data. This allows us to determine the skill content of vacancies. This step is necessary, because no textual description of the jobs is provided in the vacancy database, which would allow the skills to be measured directly. By taking the network approach, we can identify and quantify occupation-defining skills even though no direct link exists between ESCO and the Austrian occupational classification system. Unfortunately, ESCO does not provide information about the complexity of a skill or skill level, so we refer to the ILO skill-level categorisation to approximate this missing information.

The rest of the paper is structured as follows: In Sect. 2, we briefly review the related literature. The data sources used for the analysis are described in Sect. 3. In Sect. 4, we present evidence for the development of job vacancies in Austria from 2007 to 2017. The results regarding skills demanded in vacancies are presented in Sect. 5. In Sect. 6 we compare our skill level ranking approach to a wage-based occupational ranking approach, and, in Sect. 7, we discuss the findings and present our conclusion.

## Related literature

Several studies suggest that methods used to analyse polarisation patterns need further refinement. For example, Caines et al. (2017) introduce a measure of task complexity and demonstrate that, based on this measure, routine intensity does not significantly predict wages and wage growth. Furthermore, Salvatori (2018) shows that the changing the skill mix acts as an important driver for occupational polarisation in the UK. The findings of Alabdulkareem et al. (2018) support these results. These authors use network analysis methods to demonstrate that workplace skills in the USA are polarised. Their results indicate that the polarisation of workplace skills is connected to the hollowing out of the middle class. In other research, network analyses were performed on big data extracted from publications, course syllabi and job advertisements published between 2010 and 2016. The analysis results show that unique human skills, e.g. soft skills such as communication, presentation and teamwork, are needed to complement technical and engineering skills. Specifically Börner et al. (2018) use a Multivariate Hawkes Process model and construct a directed network to analyse how soft and hard skills influence each other. The authors found that specific hard skills can predict specific soft skills and vice versa. One major finding of their network analysis is that “soft and hard skills influence each other recursively in a continuous cycle” (Börner et al. 2018, p. 12636). These findings indicate that soft skills and technical data science or engineering skills are tightly connected and, in fact, that it is difficult to disentangle them. This conclusion is quite similar to one that can be drawn from a review of the economics literature on task-based technological change, namely, that jobs usually consist of a combination of many complementary abstract, manual, routine and non-routine tasks (Autor 2015).

Börner et al. (2018) present an argument regarding the complementarity of skills that is supported by the results of Deming (2017) and Grundke et al. (2018). The authors highlight the increasing importance of specific skills and skill bundles for labour market outcomes, such as wages. Deming (2017) stresses the fact that employees with a combination of social and mathematical skills receive higher rewards than those with other skills and skill combinations, but Grundke et al. (2018) show that workers with certain skills (i.e. higher levels of self-organisation and advanced numeracy) receive higher rewards in digital intensive industries compared to less digitally intensive industries. Finally, Anderson (2017) illustrates that the applicability of skills matter: Even when the returns to a diverse set of skills are higher than returns to more specialised skills, workers whose skills can be applied in many different jobs (ubiquitous skills) receive fewer rewards than workers who have combined skills that allow them to fill gaps in the labour market.

While all of these results indicate that the demand for skills is changing, the actual shift of skill demand in the recent past is quantified in few empirical studies, for example, Goos et al. (2014), but they place a focus on the currently employed.

However, only studying changes in employment shares may neglect important aspects of the actual demand for skills, as shown by Hershbein and Kahn (2018) and Deming and Kahn (2018). On the one hand, employment serves as a proxy for skill demand and skill requirements simultaneously. This is due to the fact that occupational employment is the equilibrium outcome of labour demand and labour supply, which is assumed to be inelastic in the short run. This belief is challenged by Deming and Kahn (2018), who find substantial heterogeneity in skill requirements even within narrowly defined occupations, using job vacancies provided by “Burning Glass Technologies”. On the other hand, analyses based on employment at the occupational level neither capture the heterogeneity of skill demand and skill mix changes at the intensive margin (i.e. skills required within occupations) nor measure overlapping skills (i.e. skills required by several occupations). The latter implies that shifts in skill mixes at the extensive margin (between occupations) cannot be adequately captured when employment-based approaches are taken.

Moreover, because of the increasing technological capabilities to substitute for human labour, it is crucial to understand the changing skills and skill mixes more thoroughly. Acemoglu and Restrepo (2018) point out that, even though productivity gains have positive effects due to automation, which leads to rising labour demand under certain conditions, a shortage in worker’s skills could have significant negative effects with far-reaching implications for inequality. They highlight the dangers associated with workers acquiring the wrong set of skills, and especially in the upcoming era, when the use of technologies such as artificial intelligence (AI) will become wide-spread.

Overall, the review of the relevant literature reveals the importance of conducting further research on polarisation patterns. Notably, a promising approach seems to be to shift the focus of the analysis from wages to skills and to analyse vacancies rather than employment. By taking this approach, we can—to some extent—to disentangle labour demand from labour supply forces. Due to the nature of our data, we cannot study within-occupation change; instead, we address the issue of skill shifts between occupations by considering overlapping skills and then by investigating changes in the skill structure.

## Data

For our analysis we use data from two different databases: the labour market database provided by the Public Employment Services Austria (PES) and the ESCO database provided by the European Commission. In the following section we discuss each database and its limitations.

### The labour market database

The labour market database houses a great deal of information, including information about open vacancies registered with the Public Employment Services Austria. To focus on more recent developments, we extracted data on job vacancies (i.e. stock of vacancies) from 2007 to 2017; this limited our ability to compare our results with those from studies that cite pronounced polarisation tendencies in the 1990s. However, since the time period under consideration starts around a time where “[m]ost of the old industries have been rejuvenated by the ICT revolution and are all poised to innovate” (Perez 2013, p. 13), our analysis results offer some insight into the labour market effects of more recent technological trends, such as AI and advanced robotics.

The variable “stock of vacancies” reports the number of job vacancies per occupational group registered on the record date for the observation period set.^2^ The data are reported on a monthly basis and vary considerably during the year due to variations in seasonal labour demand. Hence, to obtain comparable yearly data, the public employment service calculates and reports the arithmetic mean of the monthly values for each year. Consequently, the reported figures can be interpreted as the seasonally adjusted monthly average for every year. As the vacancies provided by the Austrian labour market database are classified according to the national classification scheme (PES-classification), we convert them to an international classification scheme [the International Standard Classification of Occupations (ISCO-08)], which is based on a correspondence table provided by the public employment service. The national classification scheme is more detailed than the lowest hierarchical level of the ISCO-08 (four-digit). For this reason, 5357 PES occupations are subsumed under 380 ISCO-08 four-digit occupation codes.

### The ESCO database

The ESCO database^3^ provides detailed descriptions of occupations and associated skills, competences and knowledge. This information is highly relevant for the development of labour market policies and vocational education schemes. It includes 2942 occupations, 13485 skills/competences and 8136 qualifications. For instance, the occupation “ICT network technicians” is characterised by ten essential skills (e.g. adjust ICT system capacity, analyse network bandwidth requirements, “create solutions to problems”, identify suppliers, use precision tools), three optional skills (e.g. migrate existing data), four areas of essential knowledge (e.g. ICT networking hardware) and nine areas of optional knowledge (e.g. electronics principles). While some of these skills are highly specific (e.g. “adjust ICT system capacity”), others are quite general (e.g. “create solutions to problems”) and are applicable in many other occupations as well. Each ESCO occupation can be mapped to one ISCO-08 four-digit occupation. This hierarchical structure provides the basis for our further analysis, as we use the ISCO-08 to link the ESCO descriptions to the vacancy data described in Sect. 3.1.

### The ILO skill levels

The International Labour Organization (ILO 2012) categorises ISCO-08 major and sub-major groups (ISCO-08 one- and two-digit codes) according to four different skill levels (Table 1): Workers in occupations at Skill Level 1 (low-skill occupations) typically perform simple and routine physical tasks and the completion of basic education may be required (ISCED-97 Level 1). At Skill Level 2 (medium(-low)-skill occupations) workers typically carry out physical and socio-cognitive tasks andthe completion of first stage secondary education (ISCED-97 Level 2) up to vocation-specific education (ISCED-97 Level 4) may be required. Occupations at Skill Level 3 (medium-high-skill occupations) are characterised by requirements to perform complex technical and practical tasks and require 1–3 years of higher education (ISCED-97 Level 5b). At Skill Level 4 (high-skill occupations), workers carry out complex problem-solving, decision-making, or creative tasks and 3–6 years of higher education (ISCED-97 Level 5a and higher) are required. Skill Level 3 and Skill Level 4 can also be summarised as high-skill occupations (International Labour Organization 2020).^4^

**Table 1:** Mapping of ISCO-08 major groups to skill levels according to International Labour Organization (2012). Source: ILO

ISCO-major group	Skill level
1 Managers^a^	3 + 4
2 Professionals	4
3 Technicians and Associate Professionals	3
4 Clerical Support Workers	2
5 Services and Sales Workers	2
6 Skilled Agricultural/Forestry/Fishery Workers	2
7 Craft and Related Trades Workers	2
8 Plant and Machine Operators, and Assemblers	2
9 Elementary Occupations	1
0 Armed Forces Occupations^b^	1 + 2 + 4

^a^Occupations of the submajor group 14 Hospitality, Retail and Other Service Managers are at Skill Level 3

^b^Each of the three submajor groups is at a different skill level

### Survey data on employment and job vacancies

To contextualise our analyses with respect to previous research on labour market polarisation and highlight evidence of potential bias within our data source, we also included data from two publicly available data sources provided by Statistics Austria in our analyses: (i) the number of employees per ISCO two-digit occupation based on the European Labour Force Survey (LFS) and (ii) data on job vacancies collected by the Job Vacancy Survey (JVS). One main advantage of these data sources is their public availability, but they also have significant drawbacks regarding time and classification consistency. First, the JVS, which was implemented in 2009, used broader occupational groups prior to 2013. Data on the one-digit level of ISCO-08 are only available from 2013 onward. Second, until 2010, the LFS data were classified according to the predecessor classification of ISCO-08, namely, ISCO-88. While a conversion between the two is feasible, ISCO-88 is seriously outdated in some areas, most notably where technological change has affected the nature of the occupations significantly. This and other limitations of the data used are discussed in the next section.

### Limitations

One limitation concerns of this study concerns the representative nature of the vacancies in the labour market database. Only $40\%$ to $60\%$ of vacancies are advertised via the public employment services. Furthermore, unlike studies based on online vacancies which are biased towards high-skill jobs (Deming and Kahn 2018; Hershbein and Kahn 2018), our study is based on the vacancies advertised at PES, which are biased towards low-skill jobs (Edelhofer and Käthe 2013). If we compare the shares of each ISCO major group of the vacancies registered with the public employments services to the vacancies collected by the Job Vacancy Survey and the number of employees (Table 2), we see that Managers and Professionals are particularly underrepresented in our main data source and that both Craft and Related Trade Workers and Elementary Occupations are overrepresented. Thus, we recognise that our data only cover parts of the Austrian labour market and especially high-skill vacancies are underrepresented. This consideration is important when comparing our results with previous findings of studies that tested the polarisation hypothesis. We try to circumvent this bias in absolute values, by examining the development of shares of vacancies (skills) by skill level. Since we look at the relative growth of different skill shares, i.e. we study changes in the job vacancy structure, this low-skill bias of the data should not dramatically affect our results as long as the degree of underrepresentation remains relatively stable. However, when we compare the PES data with the JVS data (see Table 2), we can see an increase in the coverage rate of the former; this means that the PES data have become more accurate over time as the spectrum of job vacancies increases. This aspect influenced the interpretation of our results, as the more comprehensive inclusion of high-skill vacancies could have contributed to developments in that segment. Thus, our results need to be interpreted cautiously.

**Table 2:** Shares of ISCO-08 major groups in percent in 2013 and 2017

ISCO-major groups	2013			2017		
	PES	JVS	Empl.	PES	JVS	Empl.
1 Managers	1.1	2.96	4.95	1.52	2.88	4.96
2 Professionals	5.49	11.39	18.41	5.61	13.03	20.14
3 Technicians and Associate Professionals	14.4	18.88	21.82	13.95	19.27	22.16
4 Clerical Support Workers	5.02	6.71	9.70	5.50	6.19	9.36
5 Services and Sales Workers	25.03	31.83	20.14	24.17	24.62	21.02
6 Skilled Agricultural/Forestry/Fishery Workers	0.53	0.78	–^a^	0.37	0.76	– ^a^
7 Craft and Related Trades Workers	28.4	14.66	15.18	26.15	18.61	12.76
8 Plant and Machine Operators. and Assemblers	4.52	6.54	6.91	5.55	6.24	
9 Elementary Occupations	14.39	8.27	3.25^a^	15.82	9.10	3.37^a^

Due to sampling errors some data had to be excluded (indicated with ^a^)

Data are based on the Job Vacancy Survey (JVS) provided by Statistics Austria, open vacancies registered with the Public Employment Services Austria (PES) and data on employment (Empl.) collected using the European Labour Force Survey

Another limitation concerns inconsistencies between different classifications. We lose unique information in ESCO when we connect the ESCO skills, which are linked to 2942 ESCO occupations, to the more aggregated ISCO-08 four-digit occupations. These 427 occupations are further reduced to 380 ISCO-08 four-digit occupations due to the conversion between the PES classification and ISCO-08. Moreover, to conduct analyses based on the skill level categorisation as described by Goos et al. (2014, 2009), we had to reclassify the data according to ISCO-88 (see Sect. 6). This results in the reduction of 43 ISCO-08 two-digit submajor groups to 28 ISCO-88 two-digit groups. In addition, nine ISCO-88 two-digit groups had to be omitted from the employment data set due to sampling errors.

Finally, due to the chosen time period, the results presented in this paper are not directly comparable with evidence presented in the original studies on labour market polarisation that find strong polarisation tendencies for the 1990s (Autor et al. 2003, 2008; Goos and Manning 2007; Goos et al. 2014), a time when routine-task replacing technologies started to permeate the economy.

## Are vacancies in Austria polarised?

In the short run, the number of job vacancies reflects business cycle fluctuations, i.e. during an economic downturn the stock of job vacancies decreases and during an economic upswing the stock of vacancies increases. The period of 2007 to 2017 was characterised by the financial crisis of 2007 which was followed by the Euro crisis. These business cycle trends are also visible in our vacancy data (see Table 4 in Appendix).

Another determinant of job posting behaviour is labour shortage. To determine whether the development of vacancies could be traced back to changes in labour shortages, we used a list of shortage occupations in Austria in 2016 [as defined by the CEDEFOP (2016)] and calculated the share of shortage occupations among all vacancies in our observation period. This share amounts to $5\%$ in 2007 and $6\%$ in 2017. In our opinion, the magnitude and increase of this share are negligible.

**Fig. 1:**
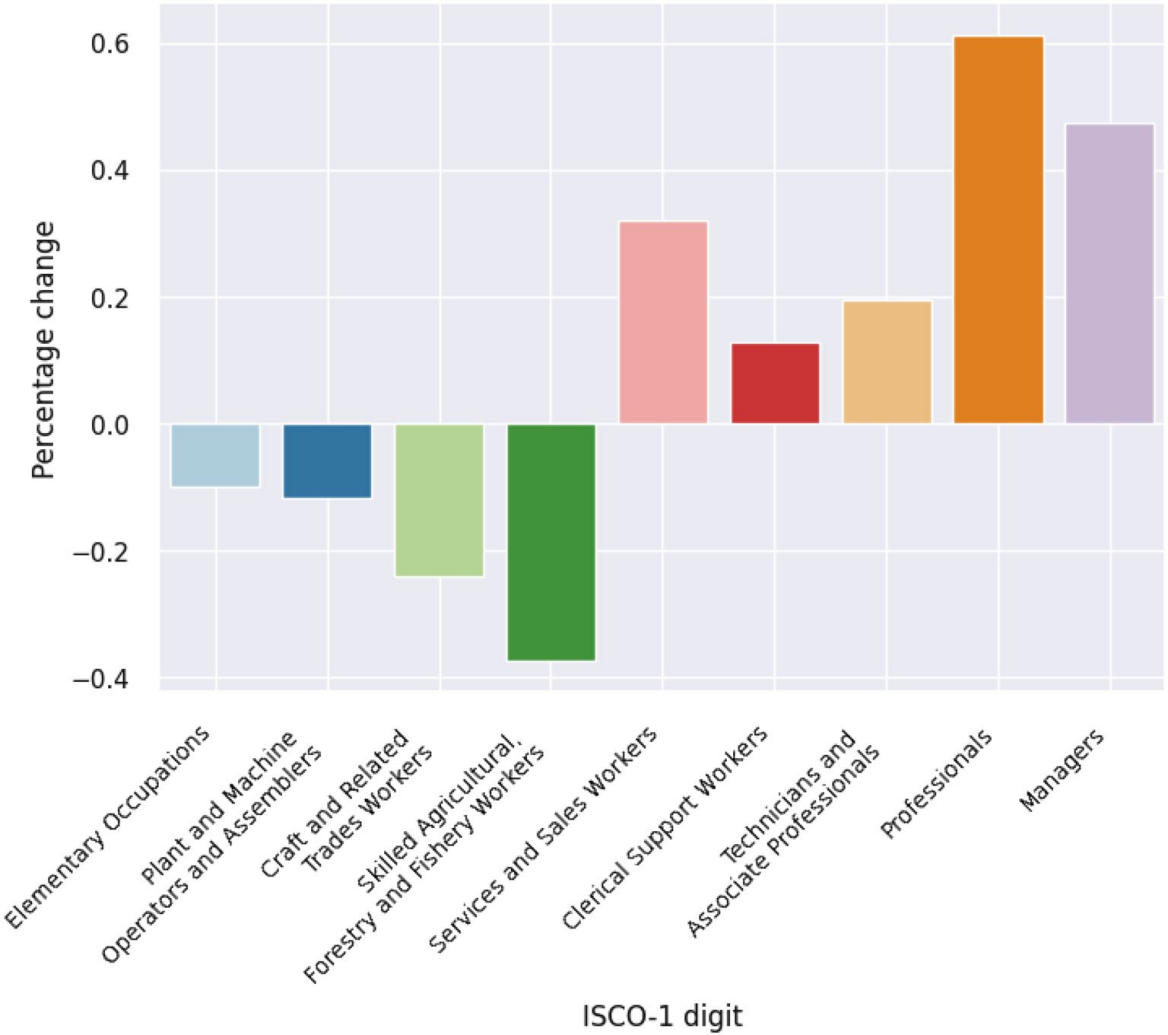
Percentage change of the shares of occupation in total vacancies by ISCO-08 1-digt from 2007 to 2017 (Source: PES)

Although these issues are interesting in their own right, we do not address them explicitly in this paper; instead we focus on our main research goal, which is to study polarisation. To achieve this goal, we use the share of each occupation ($v_i$) in total vacancies ($\kappa _i=v_i/\sum \limits _{i \in I}{v_i}$) and calculate their growth factor over the whole observation period from 2007 ($t=1$) until 2017 ($t=2$) using to Eq. 1. Figure 1 shows the percentage changes of the shares of vacancies, categorised by ISCO 1- digit major groups. While the shares of Managers, Professionals, Technicians and Associate Professionals, Clerical Support Workers and Service and Sales Workers increased, they decreased for Skilled Agricultural, Forestry and Fishery Workers, Craft and Related Trade Workers, Plant and Machine Operators and Assembler and Elementary Occupations. These results do not allow us to identify polarisation trends on the 1-digit level.

Finally, to illustrate to the extent to which vacancies in different skill level groups have gained (or lost) relative importance, we categorise the vacancies into the four skill level groups provided by the ILO classification of the ISCO major groups described in Sect. 3.3. This allows us to examine the distribution of growth factors within skill level categories. We assign each vacancy a skill level according to the ISCO major group to which it belongs. This yields 106 high-, 74 medium-high, 174 medium-low and 28 low-skill vacancies.

1 $$\begin{aligned} g_{\kappa _i}=\frac{{\overline{\kappa _{i{t_1}}}-\overline{\kappa _{i{t_2}}}}}{\overline{\kappa _{i{t_1}}}} \end{aligned}$$

**Fig. 2:**
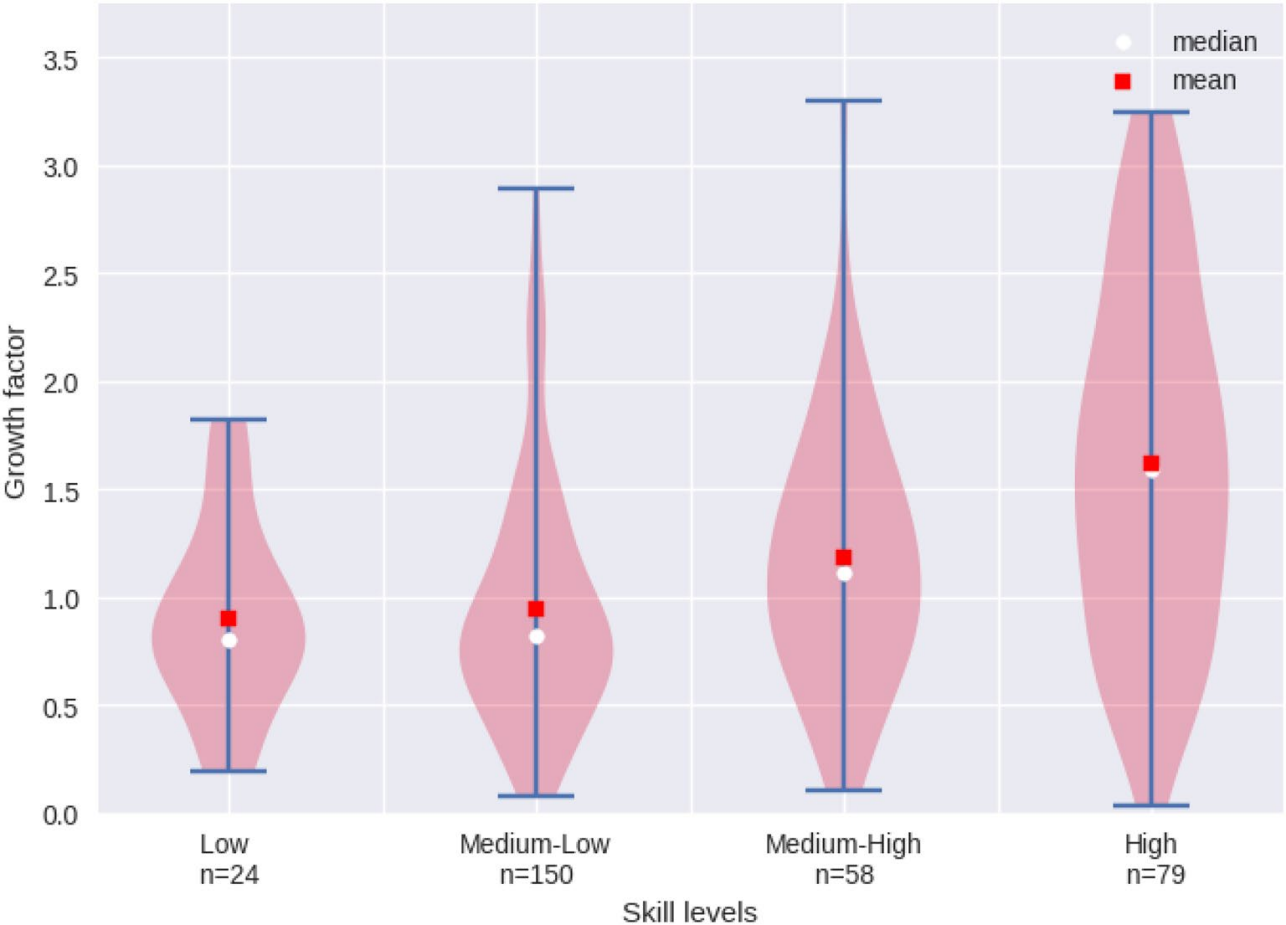
Growth factor of fraction of vacancies by skill level from 2007 to 2017 after correcting for outliers outside the 1.5 IQR. Every violin describes the distribution of the data for each skill level category. The growth factor is calculated for the shares of each occupation in total vacancies and plotted against its respective skill level, where *n* refers to the number of ISCO-08 four-digit occupations falling into the respective skill level category. The skill levels are assigned to each ISCO-08 four-digit occupation according to the ILO classification (Source: PES, ILO)

In Fig. 2, the growth factor ($g_{\kappa _i}$ on the y-axis) of each occupation share is plotted against the skill levels (x-axis) after correcting for outliers outside the 1.5 IQR. Every violin describes the distribution of the data for each skill level category. According to the polarisation hypothesis one would expect that the fraction of low-skill and high-skill vacancies has increased, while that of medium skills has decreased. However, we see in Fig. 2 that the median growth factor of the fraction of vacancies increases with the skill level. In fact, on average, the fraction of low and medium-low-skill vacancies decreased, while the fraction of medium-high-skill and high-skill vacancies increased between 2007 and 2017. Thus, we do not observe a polarisation of job vacancies (in the sense of a hollowing out of the middle-skill jobs), but rather a trend towards the growing importance of medium-high-skill and high-skill vacancies.

## From jobs to skills: a network approach

One major drawback of the occupation-based approach is that its application only gives rudimentary insights into the skills demanded in vacancies. While it provides an overview of the demand in different skill level categories, it does not provide information about the demand for skills at the extensive margin, i.e. skills that are in demand in various occupations at different skill levels. For this reason, we present an alternative approach to study skills demanded in vacancies. Since the vacancy data do not contain detailed information about the actual skills demanded, we have to rely on this alternative approach to infer the skills demanded. Using network analysis tools we can easily identify the need for skills in vacancies.

### Methods

#### From ESCO to ISCO using network analysis

**Fig. 3:**
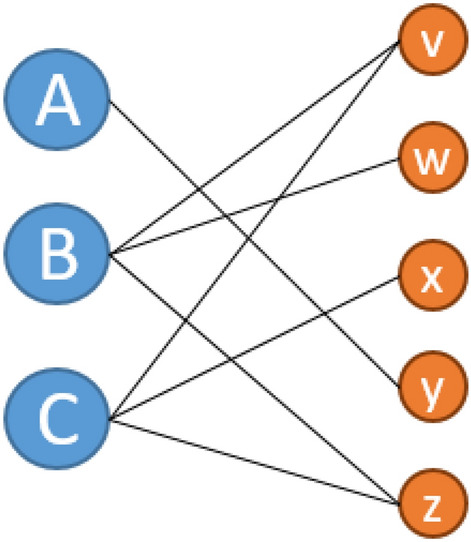
Bipartite network of jobs (uppercase letters) and skills (lowercase letters) (Source: personal illustration)

We rely on concepts and tools used in network analysis in our study. According to network theory, each *graph* or *network* consists of two different components. *Nodes* (also called *vertices*) can describe arbitrary objects, in our case, skills and occupations. These nodes are connected by links, the so-called *edges*. Here, we define that an occupation and a skill are connected if the skill is required to perform the occupation in question. If that skill is not relevant, no direct link is established between them. This leads to a network within which an occupation can be connected to several skills, and also a skill can be connected to several occupations. However, since the edges are always between an occupation and a skill, an occupation will never be directly connected to another occupation. The same is true for skills. This class of networks, which contains two distinct categories of nodes that are never directly connected, is called a *bipartite network*. Figure 3 provides an example of a bipartite network where jobs (uppercase letters) are linked to skills (lowercase letters).^5^

We use the methodology described by Alabdulkareem et al. (2018) and construct a bipartite network of skills *s* and occupations *j*, where we base the existence of an edge between *s* and *j* on ESCO *esco*(*j*, *s*).

Each essential skill, $s \in S$, is matched to occupations, $j \in J$, using $esco(j, s) \in \{0,1\}$, whereby $esco(j, s) = 1$ indicates that *s* is essential to *j* and $esco(j, s) = 0$ indicates that *s* is not required for occupation *j*. This network consists of 2937 occupations and 8258 skills linked by 46,062 edges

The hierarchical structure of ISCO-08 four-digit occupational groups, *c*, and ESCO occupations, *j* yields the bipartite network $isco(c,s)=esco(j,s)\cdot isco(c, j)$. This weighted network consists of 427 occupational groups^6^ and 8258 essential skills linked by 22,805 edges, with a median of 41 and an average of 54 skills per occupation. This approach yields ISCO-08 four-digit occupational groups which are characterised by many different skills; i.e. they are quite diverse. Consequently, we need to identify the most relevant skills, when performing these occupations.

Hence, we use the concept of revealed comparative advantage (RCA) to identify skills that are over-expressed in ISCO-08 occupational groups.^7^

2 $$\begin{aligned} rca_{isco}(c,s)=\frac{isco(c,s)/\sum \limits _{s \in S} isco(c,s)}{\sum \limits _{c \in C} isco(c,s)/\sum \limits _{c \in C,s \in S} isco(c,s)} \end{aligned}$$

We compare the relative importance of skill *s* to an occupational group (numerator in Eq. 2) to the relative importance of a skill on aggregate (denominator in Eq. 2).

Next, we construct a network/matrix of essential skills $E(c,s)=1$ with $E \in \{0,1\}$ if $rca_{isco}>1$, where an occupational group *c* relies on a skill more than expected on aggregate. This helps us to identify key occupational features and controls for ubiquitous skills (e.g. create solutions to problems). This process yields a network consisting of 8258 skills and 427 occupations linked by 22,675 edges.

#### Deriving skills demanded in vacancies

We derive the demand for skills based on the vacancy data extracted from the labour market database. After mapping the vacancies from the national classification to ISCO-08 using the official conversion of PES, we use the matrix of the monthly average of vacancies in each occupational group for every year *AV*(*y*, *c*) and the matrix of essential skills *E*(*c*, *s*) to calculate the average demand for each occupation-defining skill *s* per year in Eq. (3).

3 $$\begin{aligned} \mathbf {S(y,s)}=\mathbf {AV(y,c)} \cdot \mathbf {E(c,s)}. \end{aligned}$$

Hence, we connect the skills which were identified as occupation-specific from the RCA to the vacancies provided by the public employment services via their ISCO-08 code. This yields a weighted matrix with 380 occupations and 8258 skills. Note that *E*(*c*, *s*) is time-constant, while the weights of the occupations *AV*(*c*, *y*) vary over time due to changes in the posted vacancies.

Next, in Eq. (4), we normalise matrix *S* by the sum of the monthly averages of vacancies in each year.

4 $$\begin{aligned} \psi (v,y) = S(y,s) \cdot \sum \limits _{c \in C} AV(c,y)^{-1} \end{aligned}$$

Each element of the matrix $\psi$ represents the weight of occupation-defining skills in relation to all posted vacancies in a given year. This allows us to interpret the elements of matrix $\psi$ as the relative importance of an occupation-defining skill in a given year. Therefore, by focusing on $\psi$, we can analyse and compare the increasing or declining importance of each occupation-defining skill in the posted vacancies. However, we cannot capture any shifts in the skill-content within vacancies.

#### Skill levels

To analyse the polarisation of skills demanded in vacancies, we need to assign a skill level to each skill. Since the ESCO does not provide information on the complexity of a skill, we approximate this missing information by using the ILO skill levels of the one- and two-digit ISCO-08 occupations (summarised in Sect. 3.3). First, we re-scale the skill levels to obtain values ranging from 0 to 1, whereby Skill Level 1 (low) corresponds to the value 0 and Skill Levels 2 (medium-low), 3 (medium-high) and 4 (high) correspond to the values 0.33, 0.66 and 1, respectively. Next, each ISCO-08 four-digit occupation is categorised according to its skill level group (see also Sect. 4). Based on the skill level values (*v*) obtained for each occupation (*c*), we infer unique skill values for each skill *s* in ${\mathbf {E}}(c,s)$. Since $48 \%$ of all skills are linked to more than one ISCO-08 four-digit occupation and $70 \%$ percent of these occupations belong to more than one skill level category, we need to calculate the average skill value of each skill (*s*). We assume that all occupation-defining skills are equally important for each ISCO-08 occupation to which they are linked. Similarly, the ISCO-08 occupations to which a skill is linked to by *E*(*c*, *s*) are equally important for determining the skill level values of the respective skill. Hence, we take the arithmetic mean ($A_s$) of the skill level values (*v*) of those ISCO-08 four-digit occupations to which the respective skill is linked to by ${\mathbf {E}}(c,s)=1$, given in Eq. 5.^8^

5 $$\begin{aligned} A_s=\frac{1}{n}\sum \limits _{i=1}^n v_i \end{aligned}$$

Thus, we obtain a continuous skill value for each skill in the range of 0 to 1 ($A_s \in [0,1]$).

### Results

#### Skill demand of vacancies in Austria 2007–2017

We now deepen the analysis of skills demanded in vacancies by looking at the skill values obtained in the previous section. To do so, we use the occupation-defining essential skills identified by RCA and described in Sect. 5.1.2 and calculate the growth factor of the relative importance of skills in job vacancies ($g_\psi$ in Eq. 6) to analyse the change observed between 2007 and 2017.

6 $$\begin{aligned} g_\psi =\frac{{\overline{\psi _{t_1}}-\overline{\psi _{t_2}}}}{\overline{\psi _{t_1}}} \end{aligned}$$

**Fig. 4:**
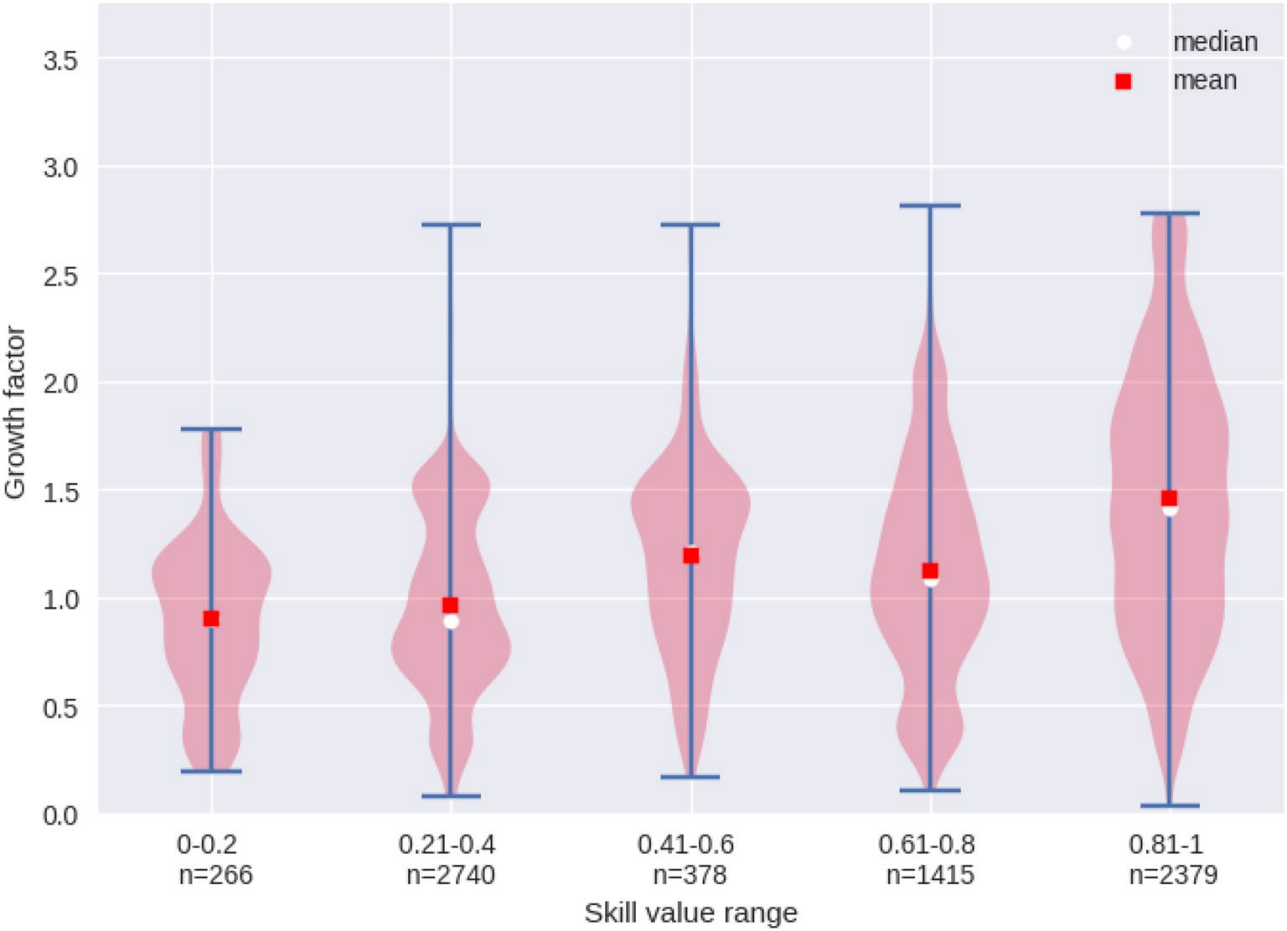
Growth factors of the relative importance of occupation-specific skills by skill value ranging from 2007 to 2017. Each violin represents the distribution of growth factors within predefined skill value ranges. The growth factor is calculated for the weight of occupation-defining skills in relation to all vacancies in a given year. The skill value ranges are calculated using network analysis methods and are based on ILO skill levels and ESCO (Source: PES, ESCO, ILO)

In Fig. 4, each violin represents the distribution of growth factors within predefined skill value ranges. Due to the fact that many skills are linked to several occupations, which fall into different skill levels, we obtain a more detailed picture of the skill content of vacancies in Austria by performing this analysis than we obtained with the analysis in Sect. 4. From these violin plots, we gain two main insights. First, the dispersion of the growth factor increases with the skill value. This implies that the development of the relative importance of occupation-defining skills in the top-skill ranges was more heterogeneous than it was for those in the bottom-skill ranges. Second, on average we observe a positive relationship between the skill value and the growth level of the relative importance of the skills. This can be seen when we compare the arithmetic mean (red square) in Fig. 4 across the skill value ranges. If we then compare the means of the growth factors, we also see that the relative importance of occupation-defining skills in the bottom skill range decreased the most, while those in the top skill range increased the most. Thus, based on these data, we do not see a polarisation pattern but rather a general trend: It became more important over this time period for applicants to have top skills in order to fill the vacancies posted vacancies posted with the public employment services.

#### Quantile regression

Thus far, we only examined descriptive evidence which suggests a positive relationship between the skill level and the growth factor of the relative importance of skills. However, due to the continuous nature of the derived skill values of the occupation-specific skills, we can also explore the relationship between changes in the relative importance of skills and their skill value more thoroughly. To do so, we use a quantile regression approach which was first introduced by Koenker and Bassett (1978). Quantile regression is used to estimate quantiles of the independent variable distribution and provide a more complete picture of the distribution than OLS, which is used to estimate the mean. As our main interest lies in studying the phenomenon of polarisation, we can use quantile regression to determine what happens at the tails of the distribution. More precisely, we can study the relationship between the skill level and the growth factor for skills at different quantiles of the change between the two observation periods. To achieve this purpose, we estimate a conditional quantile function (CQF) of the growth factor of relative importance of a skill ($g_\psi$) as a function of skill value (*s*), $Q_{g_\psi }(\tau |s) = F_{g_\psi }^{-1}(\tau |s)$ where $\tau \in [0, 1]$ denotes the quantiles (Hao and Naiman 2007). The econometric quantile regression model can then be expressed as:

7 $$\begin{aligned} y_i = \beta _0^{(\tau )} + \beta _1^{(\tau )} x_i + \epsilon _i^{(\tau )}, \end{aligned}$$

where $y_i$ is the dependent variable (i.e. the growth factor $g_\psi$ of each skill), $x_i$ is the independent variable (i.e. the skill value *s* of each skill), and $\tau$ denotes the quantiles 0.2, 0.3, 0.4, 0.5, 0.6, 0.7 and 0.8. Fitting Eq. 7 yields estimates for the seven conditional quantiles of the growth factor given the skill level (see Table 5). The coefficients are interpreted as the effect of a unit change in the dependent variable on quantiles of the distribution of the independent variable. To make the regression results more accessible, they can also be examined graphically.^9^

**Fig. 5:**
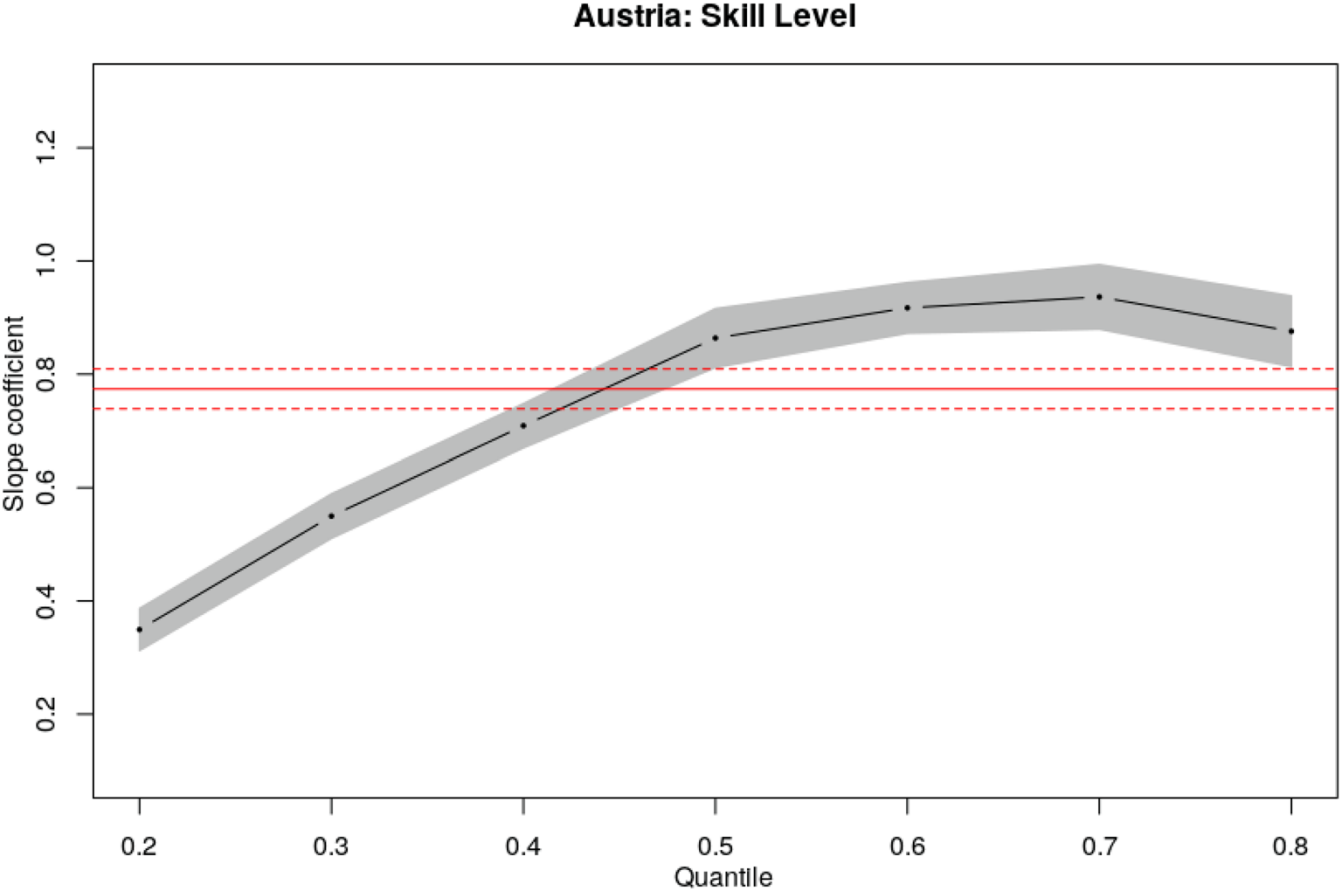
Quantile regression of growth factors of the relative importance of skills. Each dot represents the slope coefficients for the quantile indicated on the x-axis. The lines with the 95% confidence envelop connect them. The solid red line corresponds to the least square estimates and its confidence interval (dashed red lines) (Source: PES, ESCO, ILO)

In Fig. 5, each black dot represents the slope coefficient for the quantile indicated on the x-axis and the lines with the 95% confidence envelope connect them, whereas the red line corresponds to the least square estimates and its confidence interval.^10^ The graphs clearly show that the skill-value effect on the growth factor of the relative importance of skills is significantly positive. However, the skill-value effect is much lower at the lower quantiles than at the higher quantiles. But while the size of the effect increases with the skill value in absolute terms, the relative increase associated with higher skill values becomes smaller for higher quantiles of the growth factor. Put differently, the skill value effect becomes weaker in the upper half of the distribution of the growth factors. Consequently, the results from the quantile regression confirm the positive relationship between the skill value and the growth factor of the relative importance of a skill. In addition, these results reveal that the size of the slope coefficient increases as the skill value increases, albeit at a diminishing rate.

Overall, our evidence does not indicate that a polarisation of skills demanded in vacancies is occurring; instead, it highlights the shift that is taking place to assign increasing relative importance to higher skills.

## Are our results driven by our indicators?

Our approach deviates from that taken in most of the labour market polarisation literature and, when applying this approach, we did not find polarisation patterns. Thus, the question arises whether our results are driven by the approach we have chosen. More specifically, are these results driven by the analysis of the growth factor of skill shares, by the occupation-based derivation of skill values and, consequently, the skill levels, or by our data source? To address this question, we compare changes in the structure of employment, vacancies and skills. To perform this comparison, we use, on the one hand, the wage-based skill level classification described by Goos et al. (2014, 2009) and, on the other hand, the ILO skill level classification of occupations.

To follow the approach of Goos et al. (2014), we categorise the PES-vacancies and the Austrian employment data described in Sect. 3.4 according to the four lowest-paying, nine middling and eight highest-paying occupations provided by Goos et al. (2014).^11^ Moreover, using the approach described in Sect. 5.1.3, we assign the skill levels defined by these authors to the derived occupation-defining skills. Likewise, we classify the employment data according to the skill levels of the ILO classification based on the ISCO-08 two-digit submajor occupational groups described in Sect. 3.3. In order to be able to compare our categories more effectively with the three categories described by Goos et al. (2014, 2009), we reduce the number of ILO skill level categories from four to three by grouping the medium-high and high skill levels under one category (high skill). We need to perform this step with the vacancy and employment data, but not with the skill data, since these are assigned continuous skill values (see Sect. 5.1.3).

To determine whether polarisation can be observed, we calculate the percentage point changes of the shares of employment, vacancies and skills between 2007 and 2017 within the different categories (Table 3). While polarisation patterns can be detected within the categories of Goos et al. (2014, 2009), the polarisation is not evident when using the ILO categorisations. Instead, one can observe a tendency towards upskilling when the ILO categorisation is used. These findings suggest that these patterns are determined by the skill level categorisation used; interestingly, this observation was also made by Fernández-Macías (2012). In particular, he analyses the same data as used in Goos et al. (2009) and shows that the polarisation patterns are less pronounced when using a different grouping of employment shares by occupations. Moreover, Fernández-Macías (2012) presents an alternative approach to uncovering polarisation patterns in European counties using job quality tiers based on wage quintiles and education. Instead of a unified polarisation trend, he finds heterogeneous patterns of changes in the employment structure between 1995 and 2007.

**Table 3:** Initial shares and percentage point change 2007–2017 (by classification). Source: PES, ESCO, Statistics Austria

		Employment		Vacancies		Skills^a^	
		Share 2007 (in percent)	Percentage point change 2007–2017	Share 2007 (in percent)	Percentage point change 2007-2017	Share 2007 (in percent)	Percentage point change 2007–2017
Goos et al. Skill Levels	High	39.4	4.2	17.2	29.9	29.5	14.7
	Medium	34.0	− 16.7	53.2	− 16.1	45.7	− 4.7
	Low	22.3	9.3	26.8	15.9	24.4	20.5
ILO Skill Levels	High	43.6	8.3	15.6	31.9	14	22.5
	Medium	51.1	− 3.3	68.1	− 7.4	43.1	7.8
	Low	5.3	− 36.2	16.2	0.5	42.9	1.8
1.5cmILO Skill Levels	High	20.7	21.2	4.5	55.7	10.8	24.9
	Medium High	22.9	− 3.4	11.1	22.3	19.6	14.3
	Medium Low	51.1	− 3.3	68.1	− 7.4	61.4	2.8
	Low	5.3	− 36.2	16.2	0.5	8.2	1.4

^a^Skills refers to the relative importance of an occupation-defining skill in a given year

The data are categorised according to the wage-based skill level classification of Goos et al. (2014) (Goos et al. Skill Levels) and the skill levels of the ILO classification based on the ISCO-08 two-digit submajor groups (ILO Skill Levels). For comparability, the ILO skill levels are reduced to three skill levels (high, medium and low)

One drawback of Goos et al. (2009), which is also underlined by Fernández-Macías (2012), is that the wage-based skill level categories derived from 21 ISCO two-digit occupations are not detailed. As described in Sect. 5.1.2, we have applied our approach in attempt to overcome this drawback by combining information on individual skills from ESCO and skill levels based on ISCO. By taking this approach, we demonstrate that the same skill can be identified as occupation-defining for a number of different occupations that are assigned to different skill level categories. This finding is interesting in its own right, but more importantly, it highlights the importance of skill shifts at the extensive margin. These shifts have been neglected in approaches that use broadly defined occupational groups. Therefore, we believe that the derived skill values are more suitable to analyse polarisation in the given data set.

## Discussion and conclusion

In this paper, we contribute to the literature on the well-studied phenomenon of labour market polarisation. Most studies in the field of economics have placed a focus on the change of employment shares in low-skill, medium-skill and high-skill occupations, wage-based occupation classifications, or task-based classifications (routine or non-routine). While all these studies indicate that a polarisation of employment has been taking place, they disguise the heterogeneity of different skills within occupations (Deming and Kahn 2018). Moreover, a network analysis revealed the fact that the network of workplace skills itself is polarised (Alabdulkareem et al. 2018).

We extend the existing research by studying three different aspects: First, like Hershbein and Kahn (2018) and Deming and Kahn (2018), we use vacancy data as an indicator of (unmet) labour demand. Second, we employ network analysis tools proposed by Alabdulkareem et al. (2018) to identify occupation-defining skills. This identification step allows us to evaluate the changing demand for those skills. Third, instead of relying on wage-based occupation rankings to approximate skill levels, we turn to the skill-level ranking of occupations provided by the ILO. This ranking is based on the nature of the tasks performed and the minimum education requirements. Fernández-Macías (2012) also highlighted the necessity of turning to an alternative occupation ranking pointing out inconsistencies regarding wage-based polarisation analyses.

Given that technological change has been identified as the main driver of polarisation, we assume that polarisation is mainly demand-driven and, therefore, this change should be clearly visible in vacancy data. However, our approach did not allow us to identify a polarisation of vacancies in Austria; instead, we detected a trend of increasing relative importance of medium-high-skills and high-skills regarding vacancies between 2007 and 2017. Furthermore, if we compare the number of skills to the number of job vacancies, we find that the demand for skills in the higher skill value ranges (i.e. skill values greater than 0.4) grew on average more than the demand for skills in the lower skill ranges. These results are consistent with the findings of Hershbein and Kahn (2018), providing evidence for upskilling during recessions.

In addition to performing a descriptive analysis based on pre-defined skill value ranges, we exploit the continuous nature of the derived skill values by applying a quantile regression analysis. The quantile regression results reveal a statistically significant relationship between skill values and the 0.2 to 0.8-quantiles of the growth factor of skill importance. Furthermore, the size of the effect increases as the skill values rise but at a diminishing rate. Overall, our results indicate that labour demand, as represented by vacancies, is characterised by upskilling rather than polarisation. This finding could (partly) be explained by the chosen time period: While the original studies on labour market polarisation identified the strongest polarisation tendencies in the 1990s, we can examine observations for occupations that have been subject to routine-task displacement since the 1980s.

Our findings deviate from most empirical results presented in the labour market polarisation literature. For this reason, we calculate changes in the structure of employment, vacancies and skills using the wage-based skill level classification of Goos et al. (2009) and Goos et al. (2014) and compare them to the ILO skill level classification of occupations. This additional analysis provides results that show that the observation of polarisation is sensitive to the skill level classification used. However, as our disentanglement of occupation-defining skills shows, the same skill can be occupation-defining for several occupations that fall into different skill level categories. Consequently, approaches that involve the use of broadly defined occupational groups but do not take into account skill shifts at the extensive margin, may produce misleading results. Therefore, we believe that the derived skill values are more suitable to analyse shifts in labour demand in the case of our data set, which provides the number of vacancies per ISCO four-digit occupation. Nevertheless, to assess the benefits of the derived skill values, further research should be conducted to try to apply the approach presented in this paper to comparable data sets for job vacancies. As we used international classification systems – ESCO, ISCO and ILO skill levels—our method can easily be used to analyse vacancy data in other European countries.

Even though our method seems promising, some limitations regarding the data and also the methodology must be discussed. First, the PES vacancy data are biased towards low-skill vacancies. However, during the observation period, the coverage rate of the vacancy data registered at the PES increased. If we compare the structure of the vacancy data, we conclude that the low-skill bias becomes less dominant over the period. Thus, our results may partially be driven by this improvement in the coverage of high-skill vacancies. This aspect requires further investigation. Second, the skill structure within occupations changes over time but it is not possible for us to capture this change with the data used. Hence, we may actually have underestimated the extent of upskilling that took place from 2007 to 2017 if, if upskilling also occurred within occupations. Another aspect that we cannot adequately address with the available data, is related to qualitative changes in the skill structure; namely, some skills become obsolete while others emerge. Third, no table is yet available to convert ESCO occupations to PES occupations. Thus, we have to convert ESCO and PES occupations to ISCO four-digit occupations, and the latter is is a less detailed classification than both of the former. As a result, highly specific skills that are important and thus define for ESCO occupations (e.g. “supervise camp operations” for the ESCO occupation “camping ground manager”) are identified as occupation-defining skills for much broader ISCO four-digit occupations (e.g. 1439 “Services managers not elsewhere classified”). As a result, we cannot meaningfully name the actual skills that have gained in relative importance, although this would be feasible from a technical point of view if a full integration of ESCO were possible at the national level. Therefore, we lose a lot of unique information due to data aggregation.

Finally, this study allowed us to identify several interesting future research directions. While the data-driven approach presented in this paper provides evidence for the shift of relative importance towards high skills in Austrian labour demand, further research is needed to gain additional insights into the evolution of labour markets. Such research would include the complementary analysis of unemployment data to capture possible structural changes in labour supply. This would also allow us to study the extent of skill mismatch in labour markets. The network approach used by Alabdulkareem et al. (2018), which we partially applied in this work, seems promising in this respect, if ESCO is applied to European labour market data in the future. Furthermore, although our initial assumption was that technological change is the main driver of labour market polarisation, we did not attempt to conduct a causal analysis of the impact of technological change on labour demand. However, our results indicate that future research on labour market polarisation needs to extend the focus beyond employment shifts and use classifications other than wage-based skill level classifications. Specifically, this research should be extended to incorporate alternative skill level measures and other labour market indicators such as vacancies and unemployment.

## Figures and Tables

**Fig. 6:**
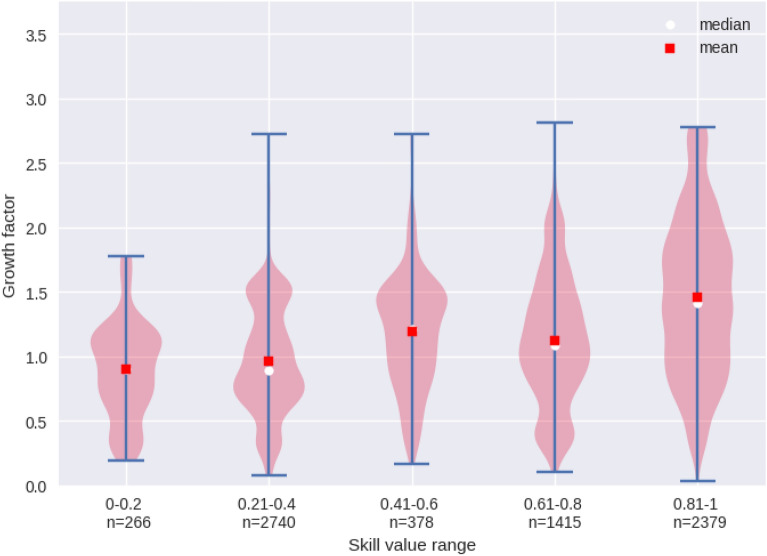
Growth factor of the relative importance of occupation-specific skills by (median) skill value range from 2007 to 2017. Each violin represents the distribution of growth factors within predefined skill value ranges.The growth factor is calculated for the weight of occupation-defining skills in relation to all vacanciesin a given year. The skill value ranges are calculated using network analysis methods and are based on ILO skill levels and ESCO (Source: PES, ESCO, ILO)

## Data Availability

The data that support the findings of this study are available from Public Employment Services Austria but restrictions apply to the availability of these data, which were used under license for the current study; thus they are not publicly available. Data are, however, available from the authors upon reasonable request and with the permission of Public Employment Services Austria.

## Appendix

See Tables 4, 5, 6; Fig. 6

**Table 4:** Monthly average stock of vacancies in Austria 2007–2017. Source: PES

Year	Vacancies
2007	37,461
2008	36,776
2009	26,736
2010	30,593
2011	31,798
2012	28,858
2013	25,833
2014	25,675
2015	28,484
2016	39,034
2017	54,477

**Table 5:** Austria: quantile regression results. Source: PES, ESCO, ILO

Quantiles	(Intercept)	Skill value
0.2	$$\underset{(~0.014)}{~0.529^{***}}$$	$$\underset{(~0.023)}{~0.349^{***}}$$
0.3	$$\underset{(~0.012)}{~0.545^{***}}$$	$$\underset{(~0.024)}{~0.550^{***}}$$
0.4	$$\underset{(~0.011)}{~0.582^{***}}$$	$$\underset{(~0.024)}{~0.709^{***}}$$
0.5	$$\underset{(~0.018)}{~0.616^{***}}$$	$$\underset{(~0.032)}{~0.864^{***}}$$
0.6	$$\underset{(~0.017)}{~0.721^{***}}$$	$$\underset{(~0.027)}{~0.917^{***}}$$
0.7	$$\underset{(~0.023)}{~0.867^{***}}$$	$$\underset{(~0.035)}{~0.937^{***}}$$
0.8	$$\underset{(~0.024)}{~1.085^{***}}$$	$$\underset{(~0.038)}{~0.876^{***}}$$

Standard errors are in parentheses

***$$p < .01$$

**Table 6:** Skill levels according to the International Labour Organization (ILO 2012).

Skill Level	Description
Level 1	Occupations at Skill Level 1 typically involve the performance of simple and routine physical or manual tasks. They may require the use of hand-held tools, such as shovels, or of simple electrical equipment, such as vacuum cleaners. They involve tasks such as cleaning; digging; lifting and carrying materials by hand; sorting, storing or assembling goods by hand (sometimes in the context of mechanized operations); operating non-motorized vehicles; and picking fruit and vegetables. Many occupations at Skill Level 1 may require physical strength and/or endurance. For some jobs basic skills in literacy and numeracy may be required. If required these skills would not be a major part of the work. For competent performance in some occupations at Skill Level 1, completion of primary education or the first stage of basic education (ISCED-97 Level 1) may be required. A short period of on-the-job training may be required for some jobs. Occupations classified at Skill Level 1 include office cleaners, freight handlers, garden labourers and kitchen assistants
Level 2	Occupations at Skill Level 2 typically involve the performance of tasks such as operating machinery and electronic equipment; driving vehicles; maintenance and repair of electrical and mechanical equipment; and manipulation, ordering and storage of information. For almost all occupations at Skill Level 2 the ability to read information such as safety instructions, to make written records of work completed, and to accurately perform simple arithmetical calculations is essential. Many occupations at this skill level require relatively advanced literacy and numeracy skills and good interpersonal communication skills. In some occupations these skills are required for a major part of the work. Many occupations at this skill level require a high level of manual dexterity. The knowledge and skills required for competent performance in occupations at Skill Level 2 are generally obtained through completion of the first stage of secondary education (ISCED-97 Level 2). Some occupations require the completion of the second stage of secondary education (ISCED-97 Level 3), which may include a significant component of specialized vocational education and on-the-job training. Some occupations require completion of vocation-specific education undertaken after completion of secondary education (ISCED-97 Level 4). In some cases experience and on-the-job training may substitute for the formal education. Occupations classified at Skill Level 2 include butchers, bus drivers, secretaries, accounts clerks, sewing machinists, dressmakers, shop sales assistants, police officers, hairdressers, building electricians and motor vehicle mechanics
Level 3	Occupations at Skill Level 3 typically involve the performance of complex technical and practical tasks that require an extensive body of factual, technical and procedural knowledge in a specialized field. Examples of specific tasks performed include: ensuring compliance with health, safety and related regulations; preparing detailed estimates of quantities and costs of materials and labour required for specific projects; coordinating, supervising, controlling and scheduling the activities of other workers; and performing technical functions in support of professionals. Occupations at this skill level generally require a high level of literacy and numeracy and well-developed interpersonal communication skills. These skills may include the ability to understand complex written material, prepare factual reports and communicate verbally in difficult circumstances. The knowledge and skills required for competent performance in occupations at Skill Level 3 are usually obtained as the result of study at a higher educational institution for a period of 1–3 years following completion of secondary education (ISCED-97 Level 5b). In some cases extensive relevant work experience and prolonged on-the-job training may substitute for the formal education. Occupations classified at Skill Level 3 include shop managers, medical laboratory technicians, legal secretaries, commercial sales representatives, diagnostic medical radiographers, computer support technicians, and broadcasting and recording technicians
Level 4	Occupations at Skill Level 4 typically involve the performance of tasks that require complex problem-solving, decision-making and creativity based on an extensive body of theoretical and factual knowledge in a specialized field. The tasks performed typically include analysis and research to extend the body of human knowledge in a particular field, diagnosis and treatment of disease, imparting knowledge to others, and design of structures or machinery and of processes for construction and production. Occupations at this skill level generally require extended levels of literacy and numeracy, sometimes at a very high level, and excellent interpersonal communication skills. These skills usually include the ability to understand complex written material and communicate complex ideas in media such as books, images, performances, reports and oral presentations. The knowledge and skills required for competent performance in occupations at Skill Level 4 are usually obtained as the result of study at a higher educational institution for a period of 3–6 years leading to the award of a first degree or higher qualification (ISCED-97 Level 5a or higher). In some cases extensive experience and on-the-job training may substitute for the formal education, or may be required in addition to formal education. In many cases appropriate formal qualifications are an essential requirement for entry to the occupation. Occupations classified at Skill Level 4 include sales and marketing managers, civil engineers, secondary school teachers, medical practitioners, musicians, operating theatre nurses and computer systems analysts

## Footnotes

1 The skill levels are operationalised based on the ISCO major group, the level of formal educational requirements for performing these tasks, the amount of informal on-the-job training and job-related experience required to perform the tasks in an occupation. See Table in Appendix  for a description of the skill levels.

2 Note that these data do not provide any information on the content of the job openings (e.g. the text of the job ads, the job advertiser), but instead reports the number of job posts for each occupational group coded according to the national classification scheme.

3 The Classification of European Skills, Competences, Qualifications and Occupations was developed by the European Commission in cooperation with the CEDEFOP and various stakeholders from 2016 until 2018.

4 Note, that (extensive) on-the-job training and (prolonged) experience can substitute for formal education in some cases at Skill Levels 2, 3 and 4.

5 Note that the network of skills and occupations could also be used to determine the skill complementarity of occupations as in Alabdulkareem et al. (2018). However, this would go beyond the scope of the paper at hand.

6 Note that not all of the 436 ISCO-08 occupations contain ESCO occupations. These are typically occupational groups with no economic activity in the EU such as “ fire wood collector”.

7 RCA has been used for different research contexts including the analysis of exports of nations Caldarelli et al. (2012), Hidalgo et al. (2007), the emergence of technology-based sectors Colombelli et al. (2014) or the role of technological relatedness for technological change Boschma et al. (2014).

8 In addition, we calculated the median skill level of each skill and obtained similar growth factors. The results are provided in Appendix.

9 The descriptive statistics and regression coefficients are provided in Appendix.

10 As most of the quantiles lie beyond the least square estimates, an OLS regression does not adequately describe the data at hand.

11 Note that Goos et al. (2014) use the ISCO-88 classification, hence, we use the conversion table provided by the ILO to reclassify our employment, vacancy and skill data according to ISCO-88. To analyse the skill levels, we decided to use the ISCO-08 classification of skill levels as this classification is highly similar to the classification of ISCO-88 skill levels (International Labour Organization 2020).
